# vClean: assessing virus sequence contamination in viral genomes

**DOI:** 10.1093/nargab/lqae185

**Published:** 2025-01-07

**Authors:** Ryota Wagatsuma, Yohei Nishikawa, Masahito Hosokawa, Haruko Takeyama

**Affiliations:** Department of Life Science and Medical Bioscience, Graduate School of Advanced Science and Engineering, Waseda University, 2-2 Wakamatsu-cho, Shinjuku-ku, Tokyo 162-8480, Japan; Computational Bio Big-Data Open Innovation Laboratory, AIST-Waseda University, 3-4-1 Okubo, Shinjuku-ku, Tokyo 169-0072, Japan; Computational Bio Big-Data Open Innovation Laboratory, AIST-Waseda University, 3-4-1 Okubo, Shinjuku-ku, Tokyo 169-0072, Japan; Research Organization for Nano & Life Innovation, Waseda University, 513 Waseda Tsurumaki-cho, Shinjuku-ku, Tokyo 162-0041, Japan; Department of Life Science and Medical Bioscience, Graduate School of Advanced Science and Engineering, Waseda University, 2-2 Wakamatsu-cho, Shinjuku-ku, Tokyo 162-8480, Japan; Computational Bio Big-Data Open Innovation Laboratory, AIST-Waseda University, 3-4-1 Okubo, Shinjuku-ku, Tokyo 169-0072, Japan; Research Organization for Nano & Life Innovation, Waseda University, 513 Waseda Tsurumaki-cho, Shinjuku-ku, Tokyo 162-0041, Japan; Institute for Advanced Research of Biosystem Dynamics, Waseda Research Institute for Science and Engineering, Graduate School of Advanced Science and Engineering, Waseda University, 3-4-1 Okubo, Shinjuku-ku, Tokyo 169-8555, Japan; Department of Life Science and Medical Bioscience, Graduate School of Advanced Science and Engineering, Waseda University, 2-2 Wakamatsu-cho, Shinjuku-ku, Tokyo 162-8480, Japan; Computational Bio Big-Data Open Innovation Laboratory, AIST-Waseda University, 3-4-1 Okubo, Shinjuku-ku, Tokyo 169-0072, Japan; Research Organization for Nano & Life Innovation, Waseda University, 513 Waseda Tsurumaki-cho, Shinjuku-ku, Tokyo 162-0041, Japan; Institute for Advanced Research of Biosystem Dynamics, Waseda Research Institute for Science and Engineering, Graduate School of Advanced Science and Engineering, Waseda University, 3-4-1 Okubo, Shinjuku-ku, Tokyo 169-8555, Japan

## Abstract

Recent advancements in viral metagenomics and single-virus genomics have improved our ability to obtain the draft genomes of environmental viruses. However, these methods can introduce virus sequence contaminations into viral genomes when short, fragmented partial sequences are present in the assembled contigs. These contaminations can lead to incorrect analyses; however, practical detection tools are lacking. In this study, we introduce vClean, a novel automated tool that detects contaminations in viral genomes. By applying machine learning to the nucleotide sequence features and gene patterns of the input viral genome, vClean could identify contaminations. Specifically, for tailed double-stranded DNA phages, we attempted accurate predictions by defining single-copy-like genes and counting their duplications. We evaluated the performance of vClean using simulated datasets derived from complete reference genomes, achieving a binary accuracy of 0.932. When vClean was applied to 4693 genomes of medium or higher quality derived from public ocean metagenomic data, 1604 genomes (34.2%) were identified as contaminated. We also demonstrated that vClean can detect contamination in single-virus genome data obtained from river water. vClean provides a new benchmark for quality control of environmental viral genomes and has the potential to become an essential tool for environmental viral genome analysis.

## Introduction

Viruses are the most abundant biological entities on Earth (1–4). Bacteriophages, in particular, have a significant impact on microbial ecosystems by lysing host bacteria (5) and affecting their biogeochemical cycles (6). The potential applications of phages in bioengineering, especially in controlling bacterial communities, further highlight their importance (7,8). Nonetheless, a significant fraction of Earth’s viral diversity remains unexplored due to the challenges in cultivating these viruses in laboratory settings (9). Consequently, metagenomics, which enables the direct exploration of environmental viral genomes, has increasingly become the primary method for many researchers (9–11).

The high diversity and intricate mosaicism of environmental viral genomes make short-read genome assembly challenging, often resulting in fragmented virus sequences (12–14). Currently, many studies represent viral genomes as single contigs obtained by metagenomic assembly. While this approach avoids the risk of sequence contamination, it often results in very low genome completeness (9–11). For instance, in the IMG/VR v4 database, a repository for uncultivated viral genomes, only 3.2% of the sequences achieved 90% completeness, as evaluated by CheckV (15). This issue has increased the focus on developing contig binning approaches, where multiple contigs from the same viral species are combined into viral metagenome-assembled genomes (vMAGs) to enhance genome completeness (16). Noteworthy tools in this approach include vRhyme (17), CoCoNet (18), PHAMB (19) and ViWrap (20). Studies on marine viral metagenomics using binning have shown a significant improvement in completeness (>30%) (17). In addition to metagenomic binning, single-virus genomics is another avenue that has also gained attention (21–24). This approach uses genomic sequences from individual viral particles, known as viral single-amplified genomes (vSAGs), obtained from samples. Some of these vSAGs consist of multiple contigs that are theoretically assumed to originate from a single viral particle, and the integration of multiple contigs promises to yield high-quality vSAGs.

However, assigning multiple contigs to single genomes carries the risk of inadvertently mixing sequences from different viruses, leading to genome contamination (25). Particularly, metagenomic binning can introduce contamination through computational processing, whereas single-virus genomics can lead to physical contamination from viral particles. Such unintentional contamination can bias subsequent analyses, including functional genomic and taxonomic assignments. As these approaches become widely accepted, it is crucial not only to improve genome completeness but also to detect contamination to prevent contaminated viral sequences from accumulating in databases and spreading inaccurate research. In bacterial and archaeal research, contamination detection primarily relies on the analysis of duplicated single-copy marker genes (26). However, for viruses, analogous strategies are less straightforward due to the absence of universal single-copy marker genes. The viral binning tool vRhyme (17) employs protein redundancy as an indicator of contamination risk. However, contamination can only be detected if homologous genes are present among contigs in identical genomes, limiting the scope of detectable contamination. Although CheckV (27) directly predicts host bacterial genome contamination, it inadequately detects virus sequence contamination and presents risks of sequence redundancy. Therefore, a comprehensive and user-friendly detection system for virus sequence contamination is required.

Here, we introduce vClean, a machine learning tool designed to assess contamination risks in viral draft genomes consisting of multiple contigs. This tool evaluates contamination risk by analysing nucleotide sequence features, including *k*-mer frequencies and patterns of gene possession, such as the duplication of newly identified single-copy-like marker genes. When vClean identifies draft genomes with a high risk of contamination, it refines them to retain only the longest contigs. To demonstrate the versatility of the tool across diverse datasets, vClean was applied to environmental vMAGs and vSAGs as well as to simulated data. vClean was shown to address contamination arising in both vMAG and vSAG construction, effectively detecting contaminants that traditional methods failed to identify. Furthermore, purifying genomes with vClean improved the precision of both phylogenetic and gene function analyses. Implemented in Python, vClean is available to be downloaded for free at https://github.com/TsumaR/vClean.

## Materials and methods

### Defining single-copy-like marker genes

We defined single-copy-like genes of double-stranded DNA (dsDNA) phages from all complete viruses classified as Caudoviricetes in the IMG/VR3 database (28) (downloaded on 12 October 2020). The 150 292 sequences, determined to be complete according to direct terminal repeats and lacking polyN sequences, were collected. Next, we employed hmmsearch from HMMER (29) v3.3.2, to identify all genes against the Pfam-A (30) v35.0 database and extracted the top hit for each protein. From these top hits, 93 Pfam entries were identified as single-copy-like genes that appeared in >500 genomes and were single copies in >99% of these genomes (Supplementary Table S1). Furthermore, among these single-copy-like genes, we grouped Pfam entries that were not detected in the same genomes but were presumed to have identical functions. These groups were treated as a single set of single-copy-like genes.

### Calculation of feature values for machine learning by vClean

vClean calculates three primary feature categories: (i) gene possession patterns; (ii) nucleotide sequence similarities between contigs; and (iii) other sample features.

### Feature calculation 1: gene possession patterns

First, when an input sample is provided, vClean predicts protein sequences using Prodigal (31) v2.6.3 (-p meta). The predicted protein sequences are compared with those in the Pfam-A database using hmmsearch. The top hit of each gene is compared to a list of single-copy-like genes, and the number of overlapping single-copy-like genes in the input sample is calculated and used as a feature value. To calculate protein redundancy, all protein sequences are clustered using mmseqs2 (32) (linclust --min-seq-id 0.5 -c 0.8 -e 0.01 --min-aln-len 50 --cluster-mode 0 --seq-id-mode 0 --alignment-mode 3 --cov-mode 5 --kmer-per-seq 75). The number of clusters containing multiple genes is defined as the redundant protein value and used as a feature value.

### Feature calculation 2: nucleotide sequence similarity

Second, vClean calculates several nucleotide features for each contig within the input sample. Initially, three sequence attributes (GC content, CpG content and GC skew) are calculated for each contig. GC content is the ratio of the number of G and C bases to the total number of bases in the contig. CpG content is the ratio of CG dinucleotides to the total length of the contig. GC skew is the ratio of the difference between the number of G and C bases to the sum of the number of G and C bases in the contig. From these three sequence attributes, the variance between contigs and the difference between the maximum and minimum values are calculated and used as the feature values.

Additionally, vClean calculates tetranucleotide frequencies (TNFs), pentanucleotide frequencies (PNFs) and codon usage frequencies (CUFs) for each contig. TNFs and PNFs are calculated using Jellyfish (33) v2.3.0. CUFs are determined by counting the 64 codons in each three-base step within the open reading frame and dividing it by the total number of observed codons for each contig. These frequencies are used as vectors for each contig. For each of these vectors, vClean calculates three features: the variance of PC1 (the first principal component) between contigs, the difference between the maximum and minimum values of PC1, and the minimum cosine similarity between contigs.

### Feature calculation 3: other sample features

vClean calculates the number of contigs and total length using SeqKit (34) v2.1.0. In addition, it estimates completeness for each contig and calculates their sum for each input sample. The number of contigs, their total length, estimated completeness and their sum are used as feature values.

### Simulation genome construction

We created simulation datasets for the training and testing of vClean. These datasets were generated in three steps: (i) collecting complete virus sequences from public databases; (ii) fragmenting the sequences; and (iii) creating contaminated sequence data by mixing the fragments.

For the training dataset, reference sequences were collected from GenBank (35) on 23 March 2023. The search used the term ‘prokaryotic virus NOT partial[filter]’. Coding sequences and partial genomes were manually excluded. Sequences >2000 bp were further refined using CheckV (27) v1.0.1, retaining only those sequences projected to be ‘complete’ according to direct terminal repeats (DTRs). For the testing dataset, the reference sequences were sourced from the October 2020 release of the IMG/VR3 database. The filtering process was like the training dataset, with only sequences predicted to have bacteria or archaea as hosts and expected to be completed according to DTRs being retained. Both datasets included dsDNA phage and single-stranded DNA (ssDNA) virus sequences.

Next, we generated two types of simulation datasets, self-sampling and cross-sampling, for the GenBank and IMG/VR3 databases, respectively. To generate the simulation datasets, we randomly fragmented the reference sequences and then randomly subsampled sequence fragments. The fragmentation process was executed using BBMap (36) shred.sh with the parameters min length = 2000, median = 20 000 and variance = 20 000. Only the fragmented sequences longer than 2000 bp were subsampled using BBMap reformat.sh to generate the baseline simulation genomes. Subsequently, we completed the simulation datasets by adding additional fragments to the baseline simulation genomes. In the process of creating self-sampling contamination datasets, each reference sequence was again fragmented by shred.sh using the parameters min length = 5000, median = 15 000 and variance = 20 000. We then randomly selected either one or two fragments per genome from these new fragment sets using SeqKit sample-n 2 options. These selected fragments were integrated into the previously generated baseline simulation genomes, completing the self-sampling contamination datasets. For the cross-sampling contamination datasets, we randomly selected either one or two fragments from the fragmented baseline simulation genomes, excluding the fragments of the target genome itself. These randomly selected fragments were then mixed into the baseline simulation genomes, completing the cross-sampling simulation datasets. As a result, 28 071 training datasets derived from GenBank and 14 412 IMG/VR3-derived testing datasets were created.

### Training machine learning models

Training datasets created from GenBank were used for training vClean. The machine learning model selection involved the consideration of 16 variants, including LightGBM (37), logistic regression and random forest classifier, facilitated by the default settings of the compare_models function in PyCaret (38). LightGBM was selected as the preferred model based on its high area under the curve (AUC).

The hyperparameters were optimized using LightGBMTunerCV in Optuna (39), conducting 5-fold cross-validation. The resultant parameters {objective: binary; metrics: AUC; random_seed: 0; verbosity: −1; boosting_type: gbdt; early_stopping_rounds: 150; feature_pre_filter: False; lambda_l1: 0.0004901673656685797; lambda_l2: 0.36341613865621336; num_leaves: 8; feature_fraction: 0.58; bagging_fraction: 1.0; bagging_freq: 0; min_child_samples: 20} were utilized for the training of five models, one for each cross-validation. vClean outputs the average of the results of the five models.

### Benchmarking performance using testing simulation datasets

We calculated the actual contamination rate for the created simulation datasets, as the contamination status is known. We defined the contamination rate for each dataset as the actual proportion of the total length occupied by contaminated sequence fragments. Additionally, the completeness of the simulation datasets was evaluated using CheckV v1.0.1. Because CheckV v1.0.1 lacks a mode for inputting bins, we incorporated 200-bp polyN links between contigs before data input.

Next, we applied vClean v0.0.0 to the testing datasets created from the IMG/VR3 database. We evaluated the accuracy of vClean by comparing its predictions of contamination with the actual contamination rates and genome completeness values of the testing datasets.

### Application of vClean to global ocean virome data and benchmarking

Metagenomic sequence reads were downloaded from Global Ocean Viromes 2.0 (GOV 2.0) datasets (10). The accession numbers of the acquired reads are summarized in Supplementary Table S2. First, we constructed bins to apply vClean. The acquired raw reads were subjected to quality filtering using fastp (40) v0.20.1 (-q 25 -r), kneaddata v0.12.0 and trimmomatic (41) v0.39. MetaSPAdes (42) v3.15.0, with default settings, was used to generate individual metagenomic contigs for each sample from quality-filtered sequence reads. For the assembled contigs, geNomad (43) v1.2.0 (end-to-end -cleanup -restart -splits 16 -min-score 0.7 -with disable-find-proviruses) was used to identify contigs of viral origin. Before applying the binning process, all predicted viral contigs >5000 bp were merged into one file. All merged contigs and quality-filtered sequence reads from each sample were used as input data for vRhyme (17) v1.2.0 and metagenomic bins were constructed. Contig dereplication was performed using the vRhyme internal process (the longest option).

Next, we applied vClean v0.0.0 to the constructed bins using default parameters. Specifically, a contamination threshold value was set to 0.6, and for bins exceeding this threshold, only the longest contig was used for analysis.

Subsequently, we performed quality evaluations and phylogenetic analyses under the following three distinct conditions: on the raw bins, on the longest contigs within each bin and on bins that had been purified using vClean v0.0.0. For quality evaluation, multiple contigs were concatenated into a single sequence using a 200-bp polyN and evaluated using the ‘end_to_end’ function of CheckV v1.0.1. Protein-coding regions were predicted using Prodigal v2.6.3, with the ‘-p meta’ option. For 1257 bins that were evaluated as higher than high-quality raw bins when using all contigs, vConTACT2 (44) v0.9.20 was run with the --rel-mode’ Diamond --db ‘ProkaryoticViralRefSeq201-Merged’ --pcs-mode MCL --vcs-mode ClusterONE option to construct a protein-sharing network, which was visualized using Cytoscape (45) v3.9.1. In addition, the bin was aligned using BLASTN (46) v2.5.0+ (-evaluation 1e^−3^ -culling_limit 1 -max_target_seqs 1) against the GenBank sequences obtained, as described above. The alignment of bins confirmed for multiple reference sequences was visualized using Clinker (47) v0.0.23.

### Application of vClean to single-virus genome data

We analysed 740 single-virus genome sequencing datasets that were previously reported and obtained from river water samples (Supplementary Table S2). Raw sequence reads were quality-filtered using fastp v0.20.1 (-q 25 --trim_poly_g --poly_g_min_len 8–l 50). The quality-filtered sequence reads were *de novo* assembled using SPAdes (48) v3.15.0 by applying the following parameters: --sc --careful -- disable-rr --disable-gzip-output. The assembled contigs were filtered to detect virus sequences using geNomad v1.2.0 (end-to-end -cleanup -restart -splits 16 -min-score 0.7 -with disable-find-proviruses).

For the 725 samples that contained viral contigs >5000 bp, contamination evaluation using vClean v0.0.0 and completeness evaluation using CheckV were performed, as for the metagenomic bin. The gene function of vSAGs was predicted using DRAM-v (49) with default parameters. Viral clustering (VC) was performed by using vConTACT2 v0.9.20 with the --rel-mode’ Diamond --db ‘None’ --pcs-mode MCL --vcs-mode ClusterONE options. Assembly graph (SPAdes output) visualization was performed using Bandage (50) v0.8.1.

## Results and discussion

### Determination of single-copy-like marker genes in viral genomes

Although viruses are known not to have any universal single-copy genes, we assumed that there are genes that are common single copies within a specific lineage (single-copy-like genes) and that these genes could serve as valuable markers for predicting contamination in draft genomes. We assigned Pfam entries to all 3 321 791 genes from the 150 292 complete-length virus sequences classified as Caudoviricetes in the IMG/VR3 database. Following approaches employed in bacterial and archaeal studies, such as those used in GTDB (51), we defined ‘single-copy-like genes’ (Supplementary Figure S1) based on the profile of the Pfam annotations existing in single/multiple copies. Single-copy-like genes included Pfam entries related to viral structures and the terminase large subunit (TerL) (Supplementary Table S1). TerL is stably conserved within dsDNA of phages and is frequently referenced in phylogenetic analyses. Multiple Pfam entries exhibit similar functions (e.g. ATPase and nuclease domains of TerL and minor capsid proteins), which rarely coexisted within the same genome sequence (Supplementary Figure S2). These findings underscore the functional consistency of the single copy-like genes identified in our study.

### vClean overview and workflow

**Figure 1.**
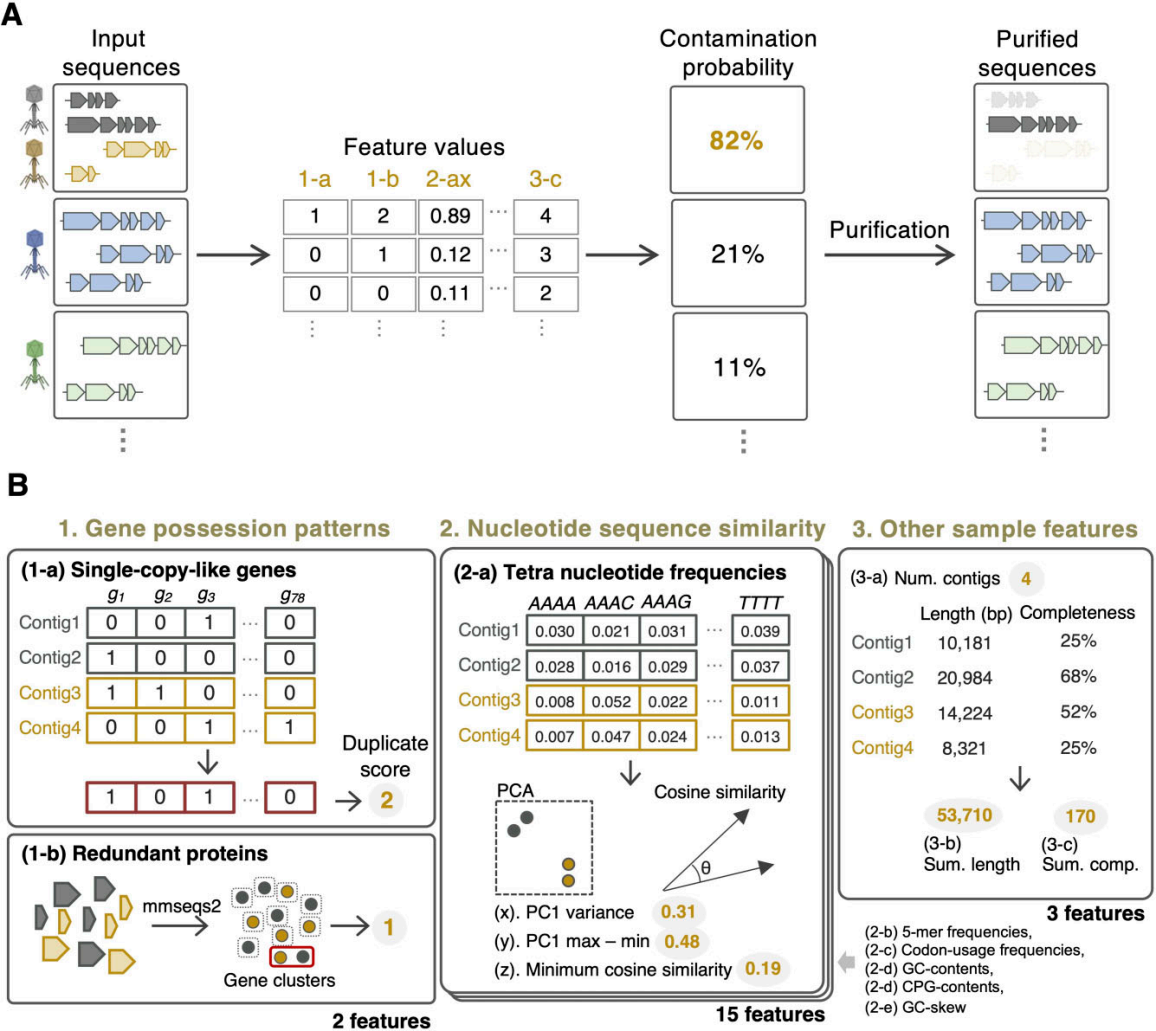
A framework for accessing virus sequence contamination from virus draft genomes. (**A**) vClean calculates 20 features and uses them to estimate contamination risk. If the contamination risk is higher than a configurable threshold value, only the longest contig is yielded as the output. If the contamination risk is lower than the threshold, all contigs are yielded as output. (**B**) Three primary categories of features used by vClean. (1-a) For the single-copy-like gene possession pattern feature, proteins encoded by the input sequences are annotated and the overlap of single-copy-like genes between contigs is counted. (1-b) For the redundant protein feature, protein clustering is performed to investigate overlaps between contigs. (2) For the nucleotide sequence similarity between contigs feature, nucleotide sequence information is extracted for each contig and differences between contigs are extracted as numerical information using multiple methods. (3) Other sample features such as total length and estimated completeness information are extracted.

vClean estimates the contamination risk of a draft genome and extracts a single contig if the risk of contamination is higher than the threshold value (Figure 1A). This process allows us to analyse high-completeness sequences with high confidence. vClean predicts contamination using three primary feature categories: (i) gene possession patterns; (ii) nucleotide sequence similarity between contigs; and (iii) other sample features (Figure 1B). For the gene possession pattern, vClean predicts contamination by identifying duplication of newly defined single-copy-like genes and the number of redundant proteins. In the second category, vClean focuses on the nucleotide sequence similarity between contigs. Specifically, vClean extracts sequence features such as *k*-mer frequencies and codon usage patterns. By calculating metrics such as the variance of PC1 (the first principal component), the maximum and minimum values of PC1, and the minimum cosine similarity from these sequence features, we converted the tables and vectors into numeric information, resulting in the creation of 15 features (Figure 1B, panel 2). The third category includes other sample features. Here, vClean incorporates three features: the total completeness value predicted by CheckV for each contig and the number of contigs. Consequently, vClean creates 20 feature values as inputs for machine learning [Supplementary Table S1 (description of all features) and Figure 1B]. vClean uses these features to predict contamination risk through LightGBM-based machine learning.

### Assessment of vClean performance on simulated genomes from IMG/VR3

To assess the accuracy of vClean, we created simulated testing data sourced from a database (IMG/VR3) that was different from the one used to source the training data. Contamination in the simulation datasets was designed based on the CheckM2 methodology (52). We created two types of contaminants, self-sampled and cross-sampled. For self-sampling contamination, we assumed the presence of identical sequence regions from closely related species in multiple copies. For cross-sampling contamination, we assumed a scenario in which sequences from distinct viral lineages were mixed. Each type represents computational contamination and physical contamination of viral particles, respectively. The simulation datasets consisted of 5556 instances of self-sampling contamination, 4432 instances of cross-sampling contamination and 4424 samples that did not include contamination from multiple contigs. The median scaffold length of the datasets was 13 786 bp.

**Figure 2.**
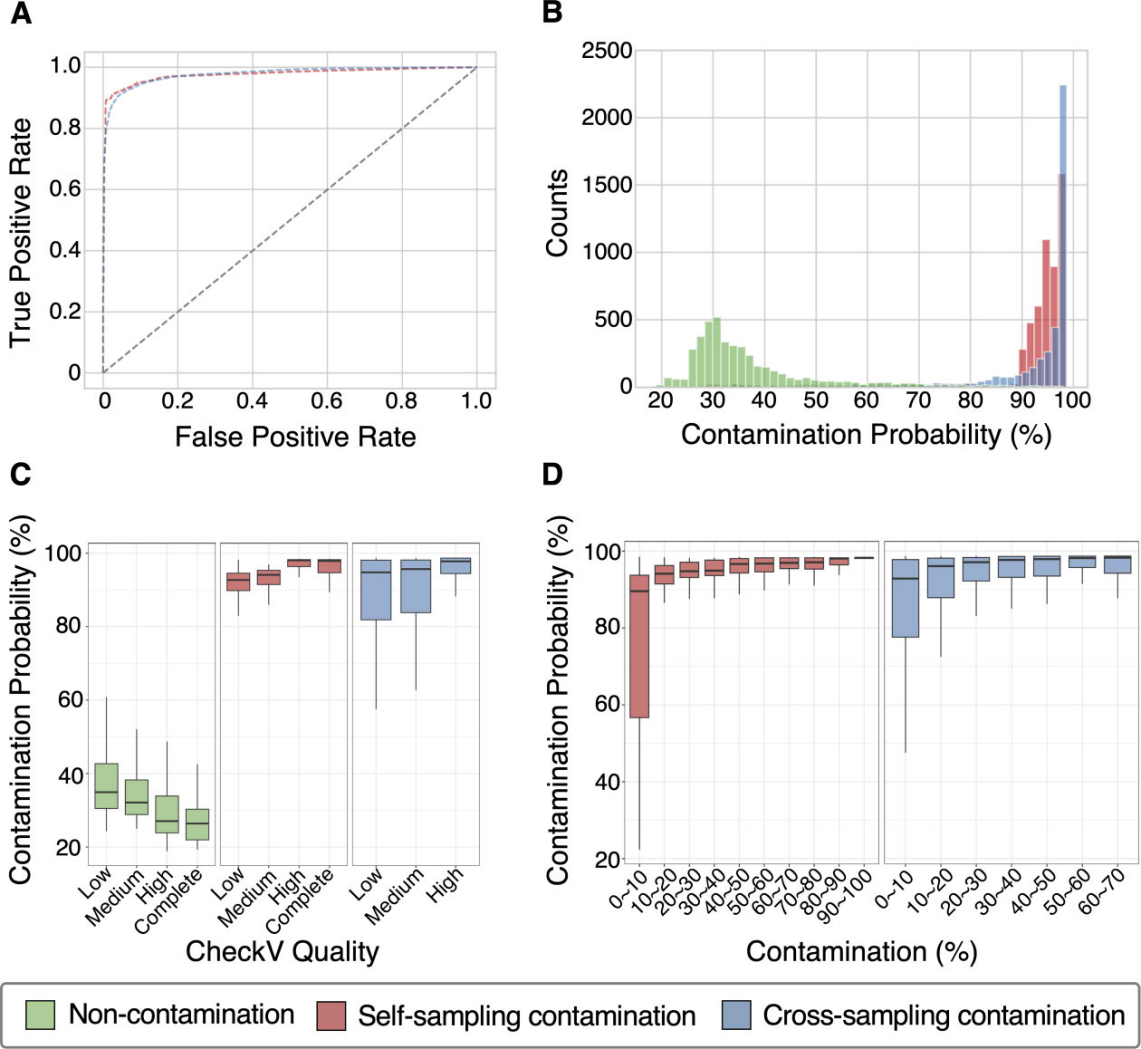
vClean prediction accuracy on the simulation dataset. (**A**) ROC curves. vClean was applied to the self-sampling contamination dataset and the cross-sampling contamination dataset derived from IMG/VR3. (**B**) A histogram of the contamination probability. (**C**) Box plots depicting the relationship between genome quality determined using CheckV (*X*-axis) and the predicted contamination probability (*Y*-axis). (**D**) Box plots representing the relationship between the actual contamination rates (*X*-axis) and the predicted contamination probability (*Y*-axis).

Subsequently, we applied vClean to the testing simulated datasets to evaluate the contamination detection accuracy. An average receiver operating characteristic (ROC) curve was generated, yielding an AUC of 0.979 (Figure 2A). This performance was substantially better than that of random predictions (AUC of 0.5), underscoring vClean’s capacity for precise contamination identification. The mean contamination probability outputs were 37.3 ± 13.5% for uncontaminated samples, 91.2 ± 18.2% for self-sampling contamination and 91.0 ± 20.6% for cross-sampling contamination (Figure 2B). Even for genomes categorized as low quality, with completeness <50% as predicted by CheckV, the uncontaminated samples had a contamination probability of 39.7 ± 13.9%, compared to 84.8 ± 17.9% and 86.1 ± 17.4% for the self-sampling and cross-sampling datasets, respectively (Figure 2C). This indicates that the prediction accuracy is maintained even for samples with insufficient genome completeness. The analysis assessed contamination probabilities for each actual proportion of contamination sequences in the simulation datasets, revealing that even at contamination rate below 10%, there were significantly higher probabilities of contamination (Figure 2D). Specifically, for self-sampling, the probability was 74.8 ± 23.1%, and for cross-sampling, it was 83.8 ± 19.6%, both markedly higher than the probabilities observed in non-contaminated samples.

By using a probability threshold of 60% to define contamination, vClean achieved an overall accuracy of 95%, which was adopted as the default setting for subsequent analyses (Supplementary Figure S3A). Using this threshold, the *F*1-scores were 0.938 and 0.927 for self-sampling and cross-sampling contamination, respectively (Supplementary Figure S3B and C). Based on our findings, we show that vClean can accurately predict both self-sampling and cross-sampling contamination with high precision. This underscores its effectiveness in detecting different types of contamination in viral draft genomes.

### Contamination detection from environmental vMAGs

**Figure 3.**
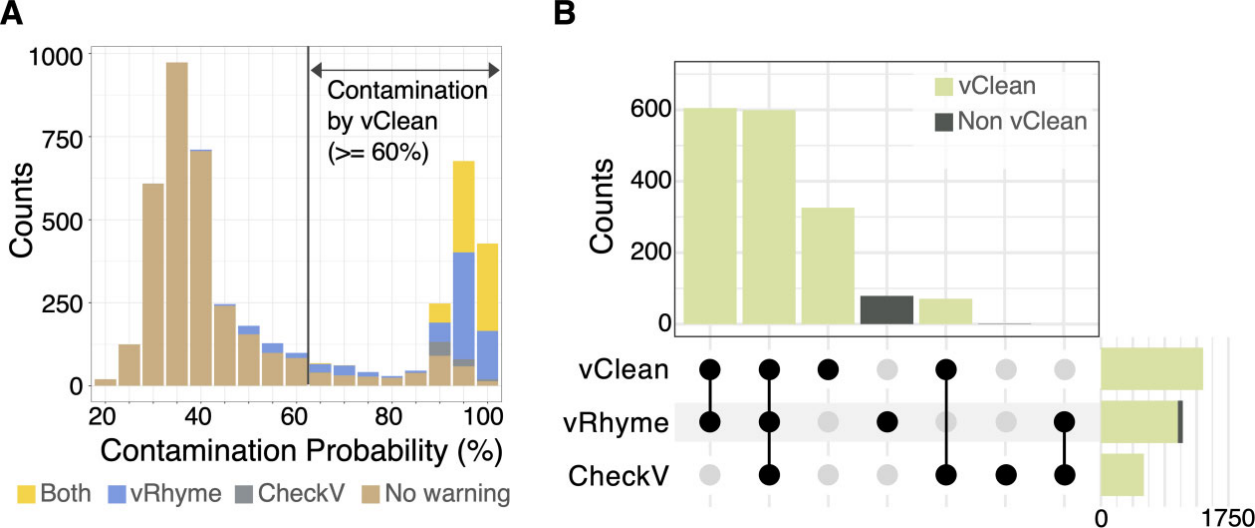
Prediction accuracy of vClean applied to metagenomic bins from marine environments. (**A**) A distribution of contamination probability for metagenomics bins with medium-quality or higher colour-coded using the classification of risk indicators detected by CheckV and vRhyme. (**B**) An upset diagram showing the number of contaminated bins detected for each analysis method. The dot plot shows the detection method, and the bar chart shows the number of contaminated samples.

We applied vClean to the vMAGs derived from marine viromes. Initially, viral contigs longer than 5000 bp, obtained from the GOV 2.0 (10), were bound using vRhyme. As a result, 42 709 contigs were binned into 13 220 bins, each containing an average of 3.23 contigs and a maximum of 43 contigs (Supplementary Table S3). vClean was applied to 4693 bins, which were suggested to have a completeness of >50% following CheckV analysis. Of these, 1604 bins (34.2%) were predicted to be contaminated. The contamination probability value plot exhibited a bimodal distribution similar to that of the simulation datasets, thus suggesting that multiple bins were contaminated (Figure 3A).

Subsequently, we compared the prediction results of vClean with two commonly used risk indicators for viral draft genomes: (i) redundant proteins from vRhyme and (ii) warnings from CheckV. Among the 4693 bins analysed, vRhyme identified redundant proteins in 1137 of them, while CheckV issued warnings for 671 bins (Figure 3B). Using the three metrics, including vClean, a total of 1681 bins were predicted to be contaminated. Of these, 95.4% were covered by vClean, with a significant observation that 326 bins (19.4%) were exclusively detected as contaminated by vClean. This outcome indicates that vClean not only encompasses contaminations detected by traditional methods but also uncovers previously unnoticed contaminations, thereby improving the sensitivity of contamination detection.

We further investigated the features of contaminants that were missed by traditional metrics. Our analysis revealed variations in sequence attributes between the contigs, including GC content, CpG content and codon usage patterns (Supplementary Figure S4). The duplication rate of our uniquely defined single-copy-like genes was 2.5% in bins deemed non-contaminated and increased to 14.3% in bins flagged as contaminated solely by vClean. This indicates that single-copy-like genes contribute to a more thorough assessment of contamination.

Furthermore, we aligned the contigs of bins to complete reference sequences in GenBank. Thirteen bins aligned with multiple reference sequences, and of these, five were identified as contaminated exclusively by vClean (Supplementary Figure S5). While viruses are known to frequently form chimeric genomes, making these alignments insufficient as direct evidence of contamination, they may represent cases where vClean detected contamination not identified by conventional methods. The discrepancy between the number of bins aligning to multiple reference sequences and the number of bins predicted as contaminated by vClean, along with the potential for chimeric genomes, highlighted the necessity for contamination detection methods like vClean that do not rely solely on direct alignment with reference sequences.

Next, we evaluated the impact of vClean on virus sequence length. We focused on 1257 bins predicted to be of high quality with a completeness of >90% when using all contigs. Out of these bins, 705 (56.1%) were predicted to be contaminated by vClean. We compared the data under three conditions: condition 1, using all bins; condition 2, analysing only the longest contig from each bin (simulating the absence of binning); and condition 3, using bins purified by vClean (Supplementary Table S4). In condition 3, only the longest contig was used for the 705 bins predicted to be contaminated. Compared to using the longest contigs (condition 2), binning combined with vClean contamination processing (condition 3) extended the average contig length by 12 401 bp and the completeness determined by CheckV increased by 18.9% (Supplementary Figure S6).

Finally, we investigated the impact of vClean on the taxonomic analysis. For conditions 1–3, we conducted a protein-sharing network analysis using vConTACT2 and clustered bins into VCs ranging from the genus to the subfamily. If contamination from different viruses persists, incorrect protein-sharing patterns between bins may arise, potentially complicating network analysis clustering. As expected, when all bins (condition 1) were used, high edge densities between bins in the network were observed, resulting in a densely aggregated plot compared with those of the reference sequences. In contrast, in conditions 2 and 3, distinct clusters representing genera and subfamilies were formed (Supplementary Figure S7). The number of sequences clustered into specific VCs increased from 737 under condition 1 to 792 under condition 3, and the average VC quality score improved from 0.39 ± 0.27 to 0.54 ± 0.27 (Supplementary Table S4). Furthermore, the number of edges between reference sequences and input sequences in the network was 2900 in condition 1, 2253 in condition 2 and 2547 in condition 3. In condition 2, connections with reference sequences were underestimated, suggesting challenges in analysing the evolutionary and community contexts of input sequences. These findings indicate that an analytical approach using vClean to decontaminate bins contributes to a more precise taxonomic prediction of virus sequences obtained from metagenomics.

### Contamination detection from environmental vSAGs

Contamination in metagenomic binning arises from the mixing of similar viruses during computational processing. In contrast, single-virus genomics carries the risk of contamination due to the physical mixing of particles. While vClean achieved accurate contamination prediction with cross-sampling contamination data, which assumes physical contamination, we tested its applicability to real single-virus genome data.

**Figure 4.**
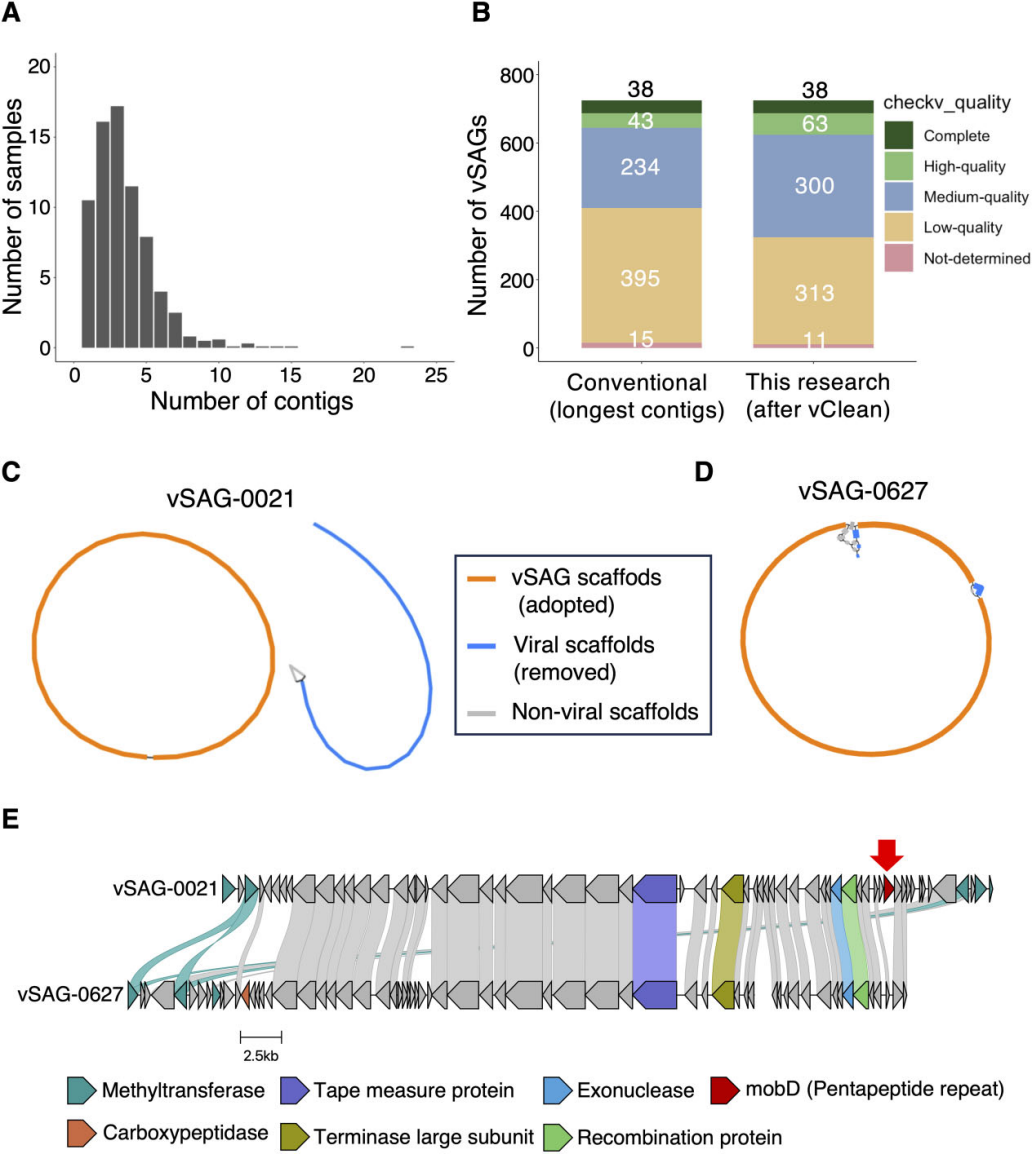
Results of vClean application to vSAGs from river water. (**A**) A histogram showing the number of contigs present in each of the 725 samples that detected at least one virus sequence. (**B**) Bar charts indicating the number of detected vSAGs, quality categorized as determined by CheckV. (C, D) Assembly graphs used in the construction of vSAGs. Graph paths are classified and displayed as scaffolds (adopted), viral scaffolds (removed), and non-viral scaffolds, as indicated in the figures. Only scaffolds that were indirectly connected to the longest contig or contigs with >5000 bp are shown: (**C**) vSAG-0021 and (**D**) vSAG-0627. (**E**) Alignment results of the gene clusters of vSAG-0021 and vSAG-0627. Edges between genes signify that the amino acid identity between the connected genes exceeds 30%. The *mobD* gene, which is unique to vSAG-0021, is highlighted by a red arrow.

We applied vClean to droplet-based single-virus genome data obtained from river water. Of the vSAGs analysed, 105 (14.2%) contained a single contig of over 5000 bp, whereas 620 (83.8%) contained multiple contigs (Supplementary Table S5 and Figure 4A). The high proportion of samples containing multiple contigs indicates the importance of integrating non-contaminated contigs for comprehensive analysis, rather than relying solely on single contig analysis, especially after identifying contamination. When comparing the use of multiple contigs identified as contamination-free for each vSAG to the use of only the longest contig, we observed a notable increase in completeness. Specifically, using vClean, the number of medium-quality or higher vSAGs increased from 315 to 401 (Figure 4B).

Next, we individually examined the contaminations detected by vClean. Using vConTACT2 to cluster vSAGs into a VC, we identified VC112, which contained two high-quality vSAGs. Among these, vSAG-0021 was contaminated, whereas vSAG-0627, which possesses two viral contigs, was assessed as uncontaminated. The assembly graph of vSAG-0021 included a closed circular scaffold and an independent ∼28 kbp viral scaffold, presumed to originate from a different virus (Figure 4C). Conversely, the assembly graph of vSAG-0627 revealed that the circular sequence primarily fragmented into two scaffolds (Figure 4D). It was confirmed that vClean could clearly distinguish between fragmentation of the same virus sequences due to assembly issues and physical contamination by different particles.

Subsequently, to confirm the advantage of using multiple fragments in the analysis, we aligned sequence fragments of vSAG-0627 with the circular contig of vSAG-0021 (Figure 4E). As a result, the fragments of vSAG-0627, which could not have been used for analysis if only the longest contig was treated, revealed two deletions of genes. One of these deletions was a *mobD* (pentapeptide repeat) gene. Notably, pentapeptide repeats mimic the DNA structures in some proteins, conferring antibiotic resistance (53–55). In the context of phages, these repeats could potentially serve as a mechanism for evading the host bacterial internal defence systems that target DNA. Without using vClean and relying solely on the longest contig for analysis, the alignment information for the region would have been missing. These results suggest that the use of vClean not only enhances the potential for detailed genomic comparisons but also provides new insights into viral evolution and host interaction mechanisms.

### Strengths and limitations

vClean is the first command-line tool capable of detecting the contamination of viral genomes originating from multiple viruses. Our validation using simulation datasets demonstrated that vClean can accurately detect contamination. Furthermore, it could predict both computational contamination from similar viruses, as expected in metagenomic binning, and physical contamination from random viruses, as expected in single-virus genomics.

However, the predictions of vClean are database dependent, which means that not all contaminants will necessarily be accurately detected. For example, single-copy-like genes have only been identified in dsDNA phages. Therefore, although single-copy-like genes are only one of several features of vClean, its detection sensitivity for contamination with closely related giant viruses or ssDNA viruses is expected to be lower than that of typical dsDNA phages in the environment. For giant viruses with genome sizes exceeding 700 kbp, which typically contain numerous contigs, manual determination of contamination and contig selection is recommended. Future developments of vClean are expected to expand the database used for training and enhance the accuracy of predictions for giant, ssDNA and RNA viruses.

Moreover, it is important to note that although the training dataset and the testing dataset were created from different databases, 92 of the 12 494 reference genomes used to create the testing dataset overlapped with the base genomes of the training dataset. However, since the fragmentation and mixing processes were conducted separately, no identical mixed data were generated in the resulting simulation data.

The single-copy genes defined in this study represent the first gene list extracted through a comprehensive analysis of viral gene copy numbers. Owing to the strict criteria used in this study, we can assert that these genes are rarely present in multiple copies of dsDNA phages. However, the biological rationale for viruses that do not maintain multiple gene copies remains largely unexplored. As the experimental verification of viral gene copy numbers progresses, it will be possible to create a more comprehensive list of single-copy-like genes, potentially improving vClean accuracy.

In conclusion, vClean represents the first unified tool for detecting viral genome contamination. This innovation allows for the confident use of multiple contigs in vMAGs and vSAGs. By leveraging vClean with metagenomic or single-virus genome data, it is feasible to construct highly reliable and complete genomes. The primary goal of vClean is to prevent inaccurate analyses. Therefore, we recommend using scaffolds that are at least 2000 bp in length, and preferably over 5000 bp, for analysis. This will allow for more accurate phylogenetic and gene content analyses, potentially having a significant impact on environmental viral genome research.

## Supplementary Material

lqae185_Supplemental_Files

## Data Availability

vClean source code is available at the GitHub repository (https://github.com/TsumaR/vClean), and also on Zenodo (https://zenodo.org/records/13928070). The simulation datasets for training and testing, along with the scripts and raw data used to obtain the results shown in this paper, can be freely accessed on Zendo (doi: 10.5281/zenodo.12549546).
