# Supplementary material for: vClean: assessing virus sequence contamination in viral genomes
Source: NAR Genom Bioinform. 2025 Jan 7;7(1):lqae185. doi: 10.1093/nargab/lqae185 (PMC11704788; doi:10.1093/nargab/lqae185)
Supplement: lqae185_Supplemental_Files [file lqae185_supplemental_files.zip › Supplementary_Data_ver24_rev2_resubmit.pdf]

SUPPLEMENTARY DATA

SUPPLEMENTARY TABLE LEGENDS

Supplementary Table S1. 93 Pfam entries identified as single-copy-like genes

Supplementary Table S2. Description of the publicly available datasets used in this study

Supplementary Table S3. Stats information for the bins created from GOV2 data

The table includes information for all 13,220 bins. It includes CheckV prediction results, vClean results, and vConTACT2 VC prediction results.

Supplementary Table S4. Comparative quantitative data for bins based on different analysis methods

From left to right, the table presents the bins constructed using vRhyme, bins from which only the longest contigs were extracted, and bins after purification using vClean. The table includes the completeness and contig length calculated by CheckV and the results from the protein-sharing network analysis using vConTACT2.

Supplementary Table S5. Stats information for the vSAGs

This table includes the number of contigs, the predicted results of vClean, and the results of vConTACT2.

SUPPLEMENTARY FIGURES

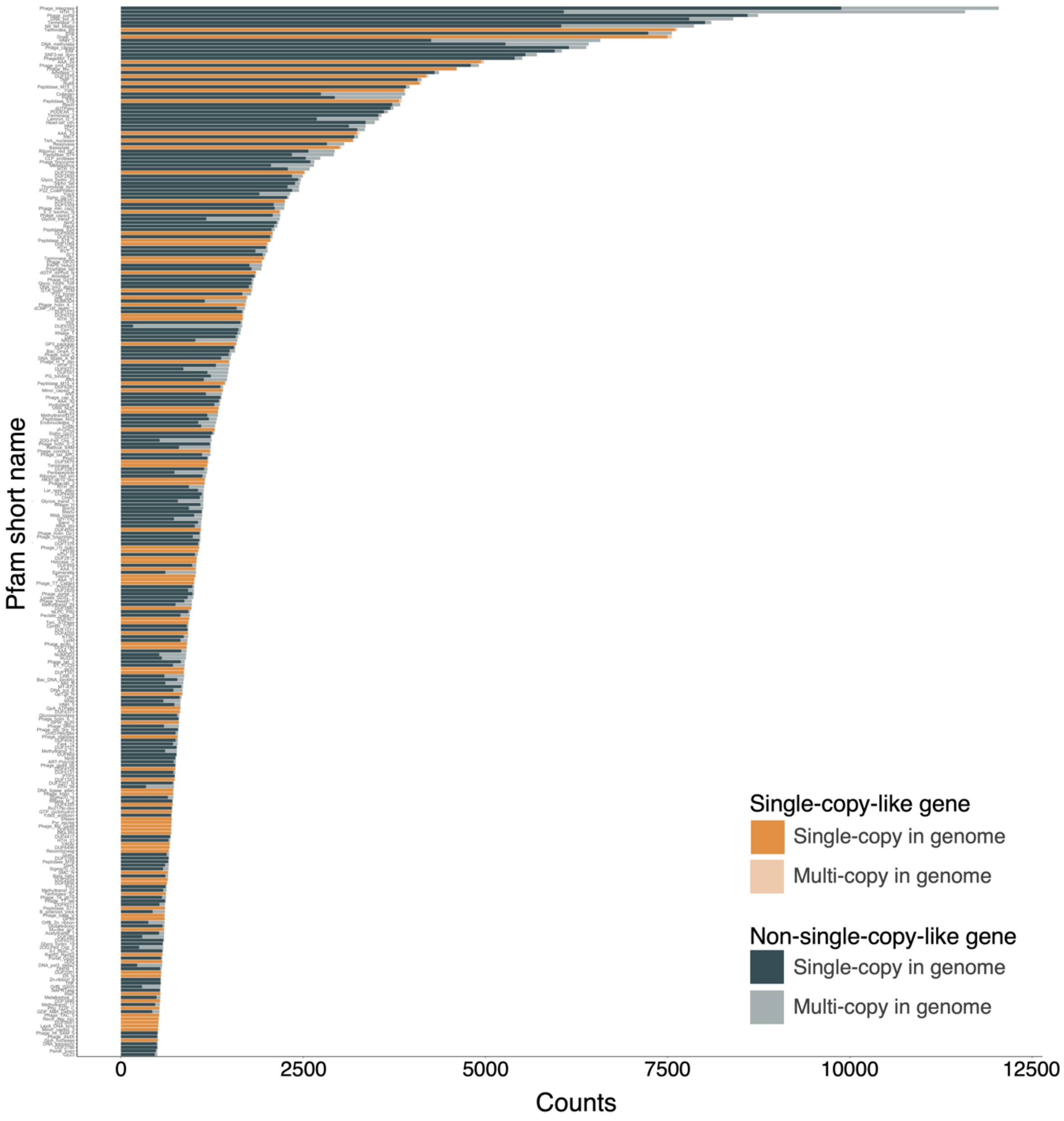

Supplementary Figure S1. Frequency distribution of Pfam entries in Caudoviricetes sequences from the IMG/VR3 database

A histogram showing the number of Caudoviricetes sequences in the IMG/VR database where specific Pfam entries were detected. This figure includes only the Pfam entries identified in more than 500 sequences. Shaded colours are used to differentiate between Pfam entries detected as single or multiple copies in each sequence. Pfam entries detected

as single-copy in >99% of the cases were defined as "single-copy-like genes" and are coloured in orange in the figure.

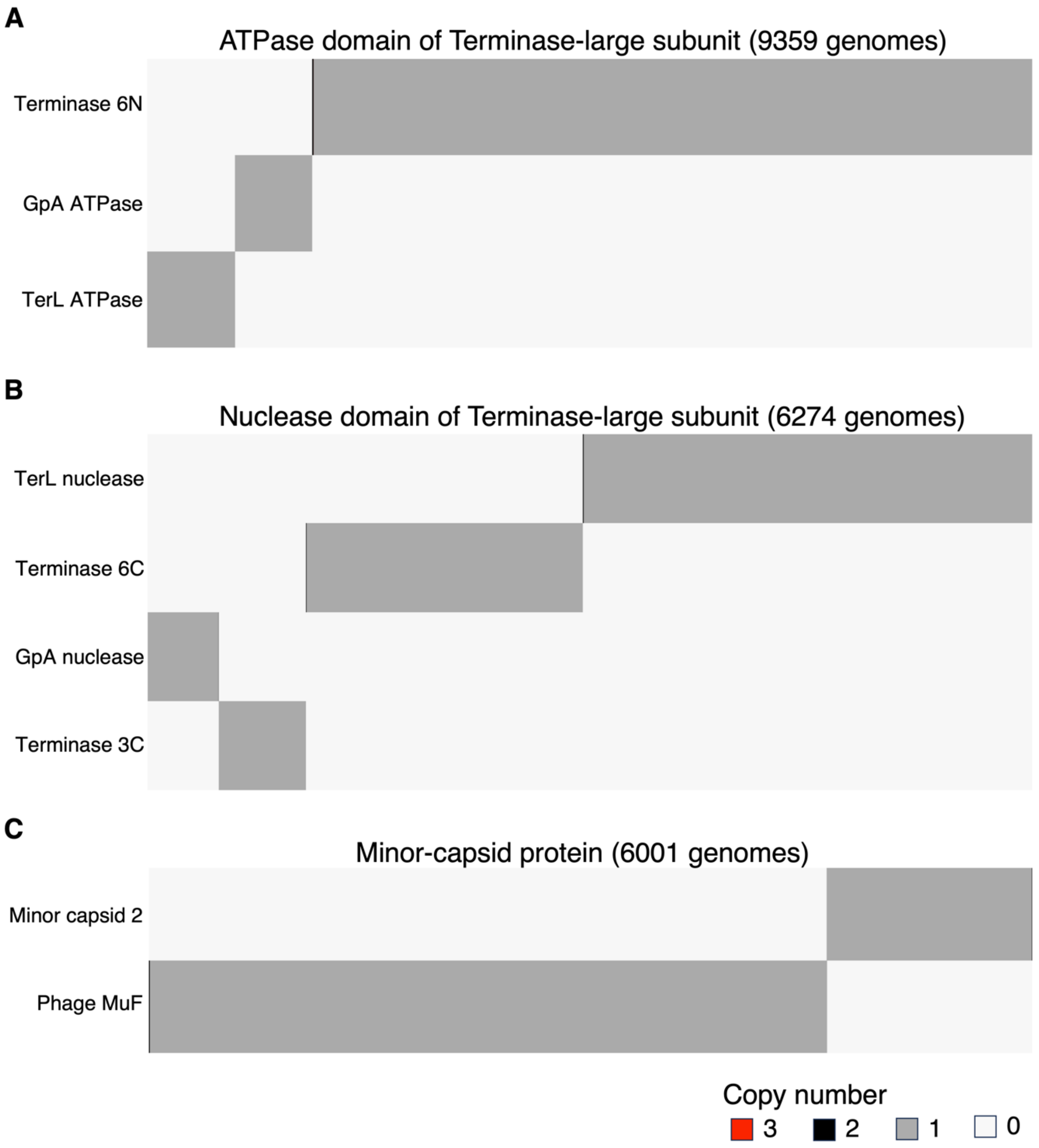

Supplementary Figure S2. Gene possession patterns of single-copy-like genes with similar functions

Heatmap of the possession patterns of single-copy-like genes related to the (A) ATPase domain of the terminase large subunit, (B) nuclease domain of the terminase large subunit, and (C) minor-capsid protein. The horizontal axis indicates genome sequences in the

database, and the vertical axis indicates each gene (Pfam entry). Only one genome sequence exhibited a copy number of three or more, specifically terminase 6N, with three copies detected. Focusing on the redundancy of similar functions, the number of sequences with duplicated similar Pfam entries was 0 for A and B and 2 (0.03%) for C.

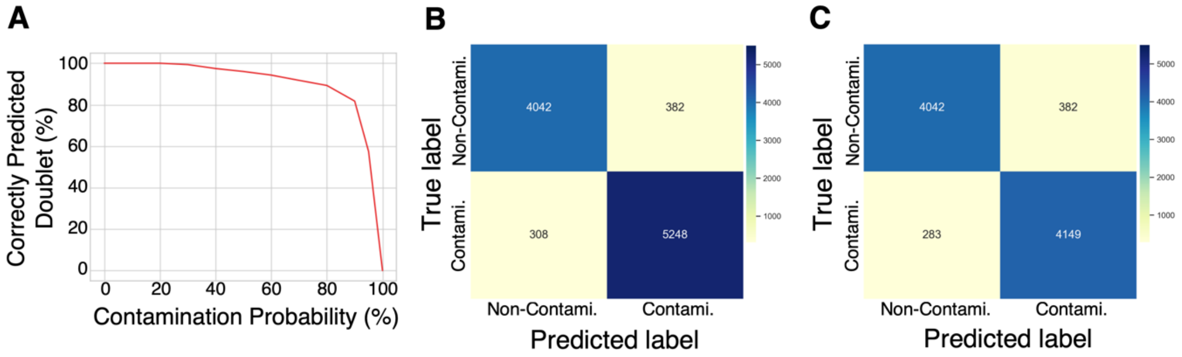

Supplementary Figure S3. Prediction sensitivity and accuracy of vClean for simulation data

(A) Sensitivity of vClean predictions as the contamination probability threshold is varied. The Y-axis represents the proportion of the contamination data that was correctly identified as contamination. (B-C) Confusion matrices showing the performance of vClean in distinguishing between (B) self-sampling contaminated and (C) cross-sampling contaminated vs. non-contaminated simulation genomes during cross-validation tests.

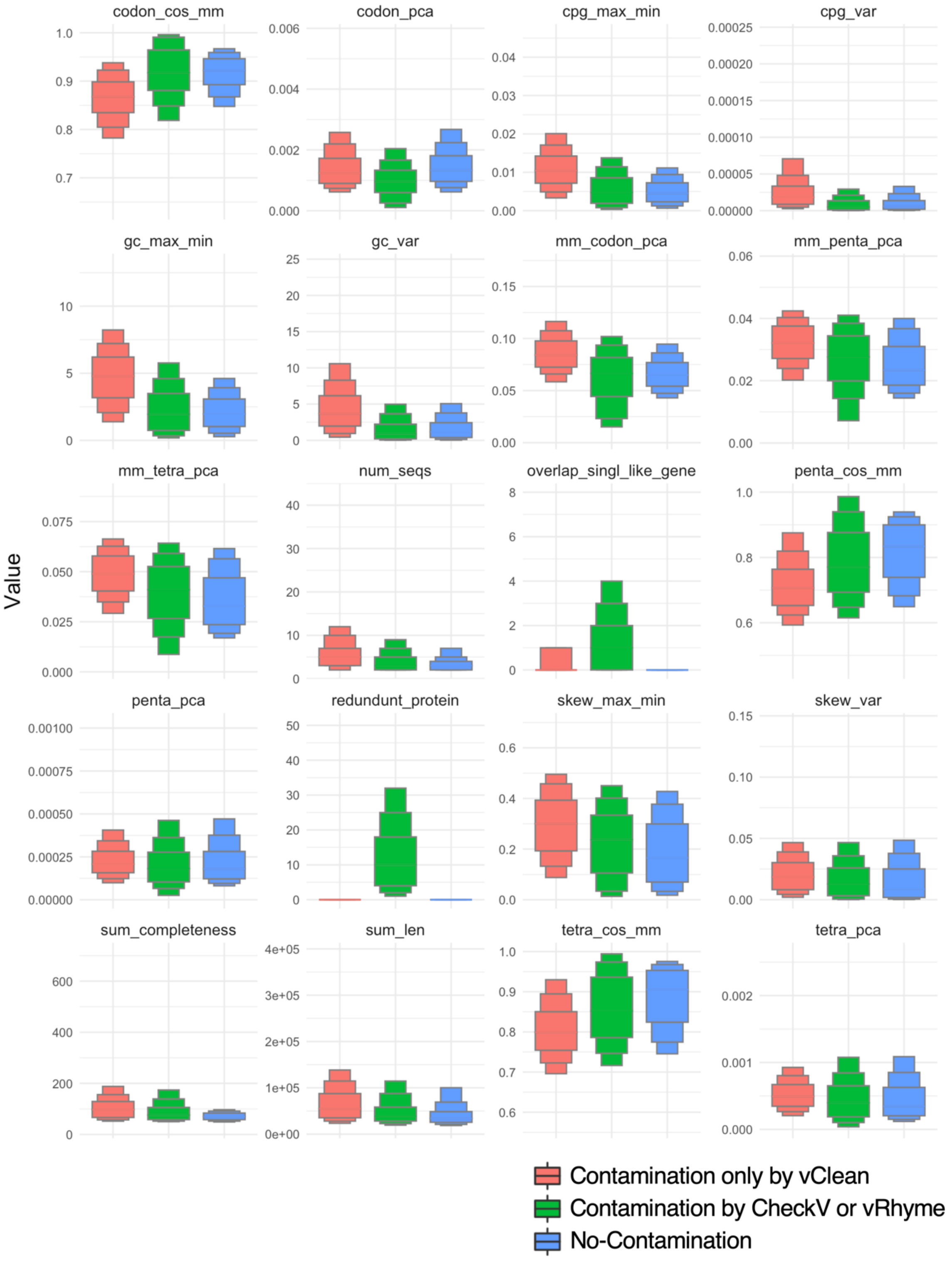

Supplementary Figure S4. Feature value distribution for bin contamination prediction types

Set of 20 boxen plots, each representing a distinct feature value for vClean. The X-axis indicates the contamination prediction status for bins, and the Y-axis displays the associated feature values.

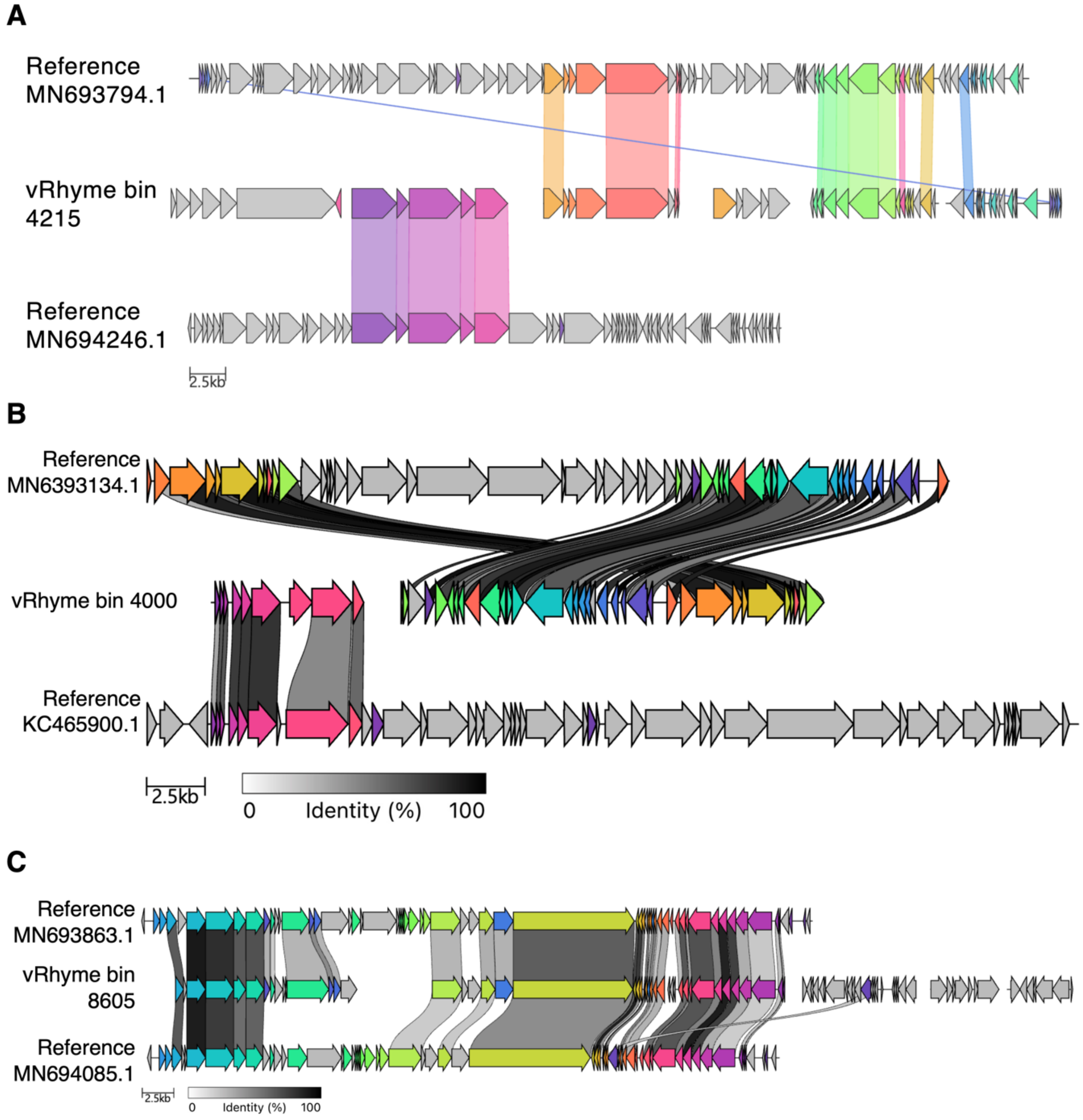

Supplementary Figure S5. Comparison of contamination bins detected by vClean with reference sequences

Synteny maps of gene clusters from vRhyme bins and viral reference sequences from GenBank. Each arrow represents a gene, with connections indicating over 80% amino acid identity. Different arrow colours indicate different protein groups. (A) vRhyme bin-4215

contains contigs that are similar to different reference sequences. (B) vRhyme bin-4000, (C) vRhyme bin 8605

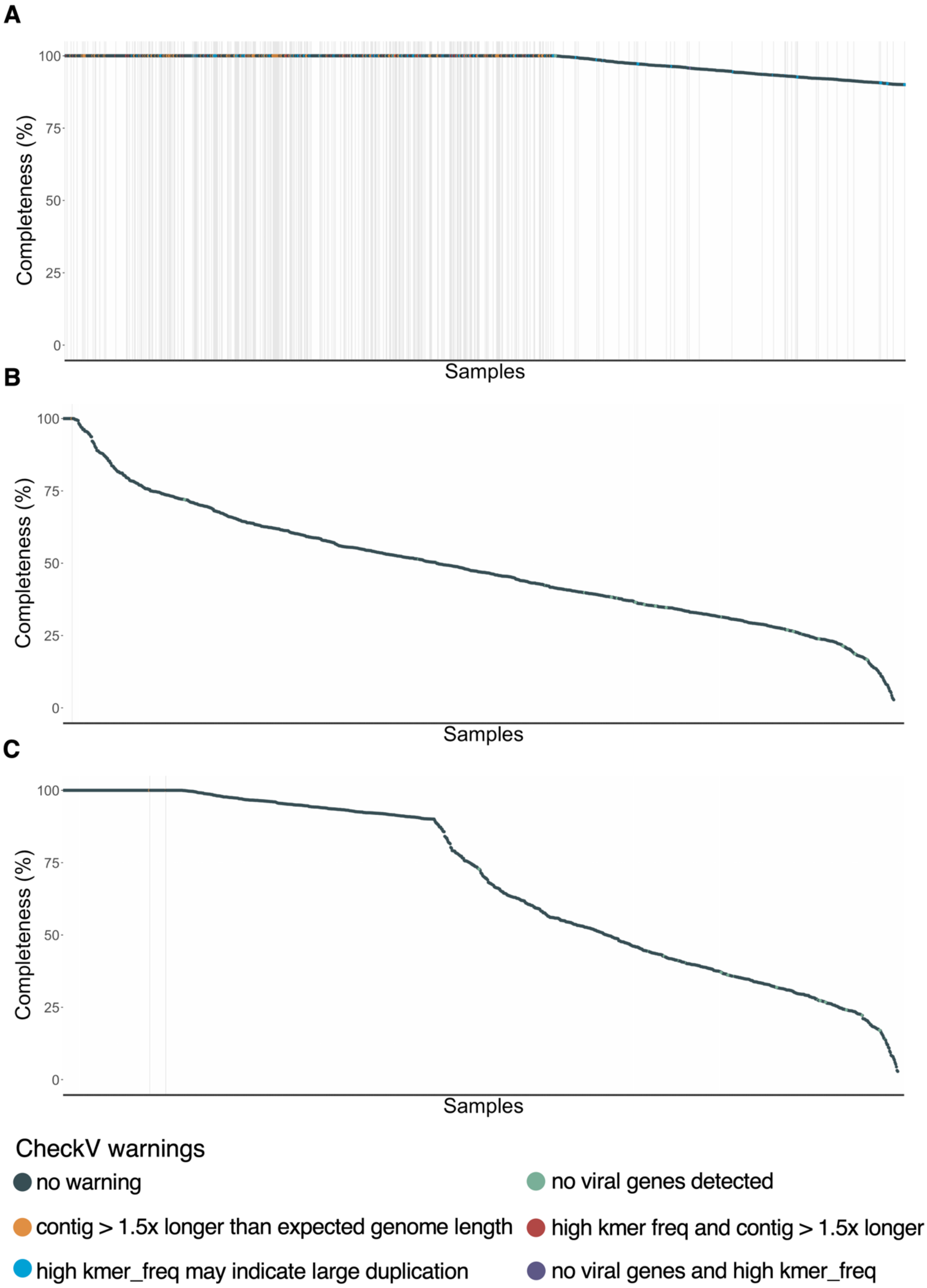

Supplementary Figure S6. Completeness comparison for each constructed bin handling method

Scatter plot illustrating the completeness of the 1,257 samples estimated using CheckV under three conditions. (A) condition1, using all bins; (B) condition 2, analysing only the longest contig from each bin (simulating the absence of binning) were extracted; (C) condition 3, using bins purified by vClean. The X-axis represents the samples, and the Y-axis depicts completeness, as estimated by CheckV. Samples with warnings other than “no viral genes detected” identified by CheckV are labelled with vertical black lines.

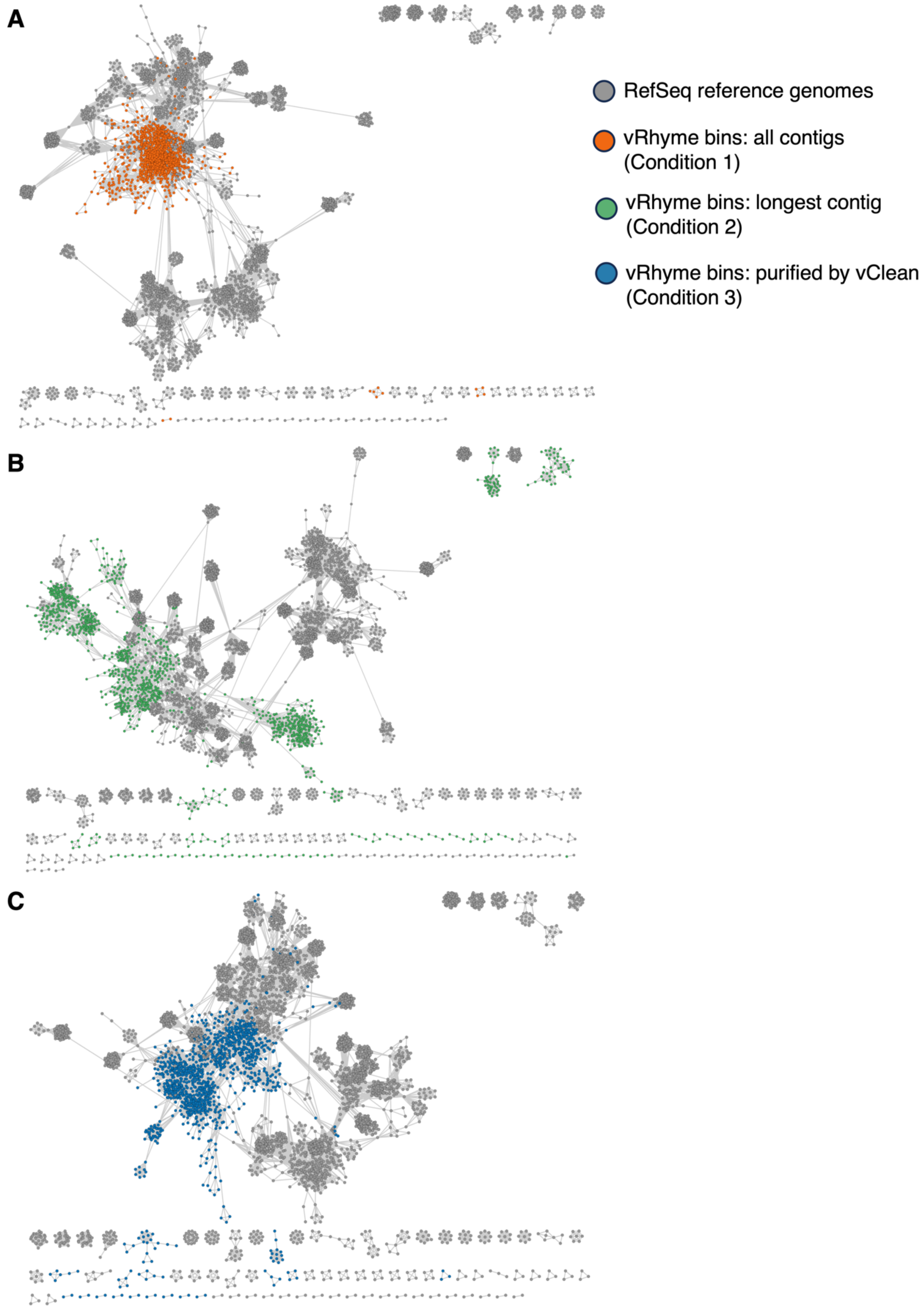

Supplementary Figure S7. Protein sharing network diagrams for each viral metagenomic bin handling method

(A-C) Protein-sharing networks for marine viral metagenomic bins, where each node represents a viral genome. Nodes are clustered together based on protein similarity and number of shared proteins. (A) condition1, using all bins; (B) condition 2, analysing only the longest contig from each bin (simulating the absence of binning) were extracted; (C) condition 3, using bins purified by vClean. The gray dots represent RefSeq reference sequences.
